# From clinical phenotype to proteinopathy: molecular neuroimaging in neurodegenerative dementias

**DOI:** 10.1590/0004-282X-ANP-2022-S138

**Published:** 2022-08-12

**Authors:** Adalberto Studart-Neto, Artur Martins Coutinho

**Affiliations:** 1 Universidade de São Paulo, Faculdade de Medicina, Hospital das Clínicas, Departamento de Neurologia, São Paulo, SP, Brazil.; 2 Universidade de São Paulo, Faculdade de Medicina, Hospital das Clínicas, Departamento de Radiologia e Oncologia, Divisão e Laboratório de Medicina Nuclear (LIM 43), São Paulo, SP, Brazil.

**Keywords:** Dementia, Positron-Emission Tomography, Amyloid, tau Proteins, Alzheimer Disease, Demência, Tomografia por Emissão de Pósitrons, Amiloide, Proteínas tau, Doença de Alzheimer

## Abstract

Neurodegenerative dementias are characterized by the abnormal accumulation of misfolded proteins. However, its diagnostic criteria are still based on the clinical phenotype. The development of biomarkers allowed in vivo detection of pathophysiological processes. This article aims to make a non-systematic review of the use of molecular neuroimaging as a biomarker. Molecular neuroimaging is based on the use of radiotracers for image acquisition. The radiotracer most used in PET is ^18^F-fluorodeoxyglucose (FDG), with which it is possible to study the regional brain glucose metabolism. The pattern of regional hypometabolism provides neuroanatomical information on the neurodegenerative process, which, in turn, has a good specificity for each type of proteinopathy. FDG is very useful in the differential diagnosis of neurodegenerative dementias through the regional pattern of involvement, including dementia with Lewy bodies and the spectrum of frontotemporal dementia. More recently, radiotracers with specific ligands to some of the pathological proteins have been developed. Pittsburgh compound B (PIB) labeled with ^11^C and the ligands that use ^18^F (florbetapir, florbetaben and flutemetamol) are the most used radiotracers for the detection of insoluble β-amyloid peptide in Alzheimer's disease (AD). A first generation of ligands for tau protein has been developed, but it has some affinity for other non-tau protein aggregates. A second generation has the advantage of having a higher affinity for hyperphosphorylated tau protein, including in primary tauopathies.

## RESUMO

As demências neurodegenerativas caracterizam-se pelo acúmulo anormal de proteínas mal dobradas. Entretanto, os seus critérios diagnósticos ainda se baseiam no fenótipo clínico. O desenvolvimento de biomarcadores permitiu a detecção *in vivo* do processo fisiopatológico. O objetivo deste artigo é fazer uma revisão não-sistemática sobre o papel da neuroimagem molecular como biomarcador. A neuroimagem molecular baseia-se no uso de radiotraçadores para aquisição da imagem. O mais usado no PET é o ^18^F-fluorodeoxiglicose (FDG), com o qual é possível estudar o metabolismo regional de glicose cerebral. O padrão de hipometabolismo regional fornece uma informação neuroanatômica do processo neurodegenerativo que, por sua vez, tem uma boa especificidade para cada tipo de proteinopatia. O PET-FDG é muito útil no diagnóstico diferencial das demências neurodegenerativas através do padrão de acometimento regional, incluindo a demência com corpos de Lewy e o espectro das demências frontotemporais. Mais recentemente, radiotraçadores com ligantes específicos a algumas das proteínas patológicas têm sido desenvolvidos. O composto B de Pittsburgh (PIB) com ^11^C e os ligantes dos que usam ^18^F (florbetapir, florbetaben e flutemetamol) são os radiotraçadores mais usados para a detecção de peptídeo β-amiloide insolúvel na doença de Alzheimer (DA). Uma primeira geração de ligantes para proteína tau foi desenvolvida, mas apresenta alguma afinidade a outros agregados proteicos não-tau. Uma segunda geração tem a vantagem de apresentar uma maior afinidade à proteína tau hiperfosforilada, incluindo nas taupatias primárias.

## INTRODUCTION

**Figure 1.**
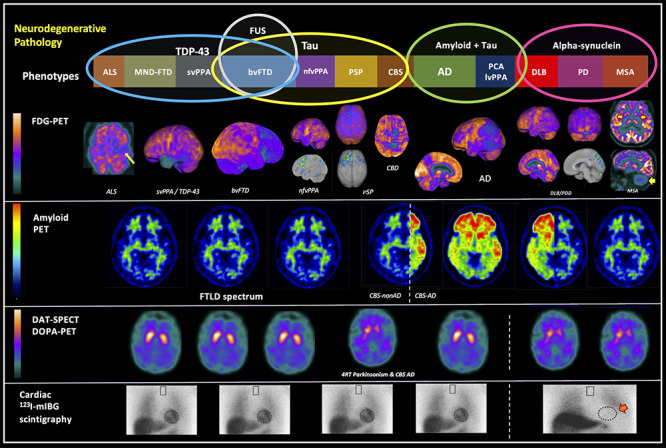
Summary of the molecular imaging findings in the different neurodegenerative diseases. **First line:** spectrum of proteinopathies causing neurodegenerative dementias and their respective clinical phenotypes. Pathological proteins: TDP-43, FUS, Tau, beta-amyloid, and alpha-synuclein. **Second line:** Patterns of regional glucose hypometabolism on brain FDG-PET that suggest or support each proteinopathy.;**Third line:** Patterns of amyloid PET with ^11^C-PIB in each condition. **Fourth line:** Patterns of uptake in dopamine transporter (DAT) SPECT or PET tracers or ^18^F-Fluordopa PET (SPECT images with ^99m^Tc-TRODAT). Fifth line: Patterns of uptake in cardiothoracic ^123^I-mIBG scintigraphy, a tracer of** **the sympathetic innervation. The white arrow in the first image on the left, third line indicates hypometabolism in the left motor cortex in motor neuron disease (ALS/Mills Syndrome); yellow arrows in the last images on the right, third line: hypometabolism in both putamen and the cerebellum seen in MSA. Orange arrow in the last image on the right in the fifth line: absence of myocardial uptake of ^123^I-mIBG seen in synucleinopathies.

Throughout the 20th century, neurodegenerative dementias were initially described based on their phenotypes and post-mortem pathological studies. In these studies, in addition to the findings of neuronal loss and brain atrophy, typical alterations of each disease were described, such as the senile plaques and neurofibrillary tangles described by Alois Alzheimer^1^, the intracellular eosinophilic inclusions observed by Fritz Heinrich Lewy^2^ and the intraneuronal argyrophilic inclusions reported by Arnold Pick^3^. Only in the last decades, with the development of immunohistochemical methods, it was discovered that these pathologies are due to the intra- or extracellular accumulation of misfolded proteins^4^. Therefore, neurodegenerative dementias could be classified by both clinical syndromes and underlying proteinopathies (Figure 1).

FTD: frontotemporal dementia; ALS: amyotrophic lateral sclerosis; MND: motor neuron disease; svPPA: Semantic variant primary progressive aphasia; nfvPPA: Nonfluent (or agrammatic) variant primary progressive aphasia; CBD: corticobasal degeneration; CBS: corticobasal syndrome; PSP: progressive supranuclear palsy; AD: Alzheimer's disease; lvPPA: Logopenic variant primary progressive aphasia; DLB: dementia with Lewy bodies; PD: Parkinson's disease; PDD: Parkinson's disease with dementia; MSA: Multiple Systems Atrophy; PET: Positron Emission Tomography; SPECT: single photon emission tomography; FDG: ^18^F-Fluorodeoxyglucose; PIB: Pittsburgh Compound-B labeled with carbon-11.

Despite advances in the pathological characterization of neurodegenerative dementias, its diagnostic criteria are still based on clinical phenotypes and not on proteinopathies^4^. However, this leads to some problems^4^ ^,^ ^5^. First, the accuracy of the clinical diagnosis of degenerative dementias is not adequate. Second, a single proteinopathy can lead to different phenotypes (for example, Alzheimer Disease (AD) can manifest as several variants: amnestic, aphasic or logopenic, visuospatial, dysexecutive and behavioral)^5^. The opposite also occurs: different proteinopathies can lead to the same phenotype, as is the case with the behavioral variant of frontotemporal dementia (vbFTD)^5^. Third, diagnostic criteria are not very sensitive to detect preclinical or prodromal stages. Finally, efforts in the development of disease-modifying treatments have been directed towards drugs that target these pathological proteins. 

For all these reasons, in recent decades biomarkers have been developed to enable in vivo detection of the pathophysiological process, thus providing a better understanding of the natural history, an improvement in the sensitivity and specificity of the diagnostic criteria, and the development of disease-modifying treatments^6^ ^-^ ^9^. This article aims to conduct a non-systematic review of the literature on the use of molecular neuroimaging as a biomarker of the main neurodegenerative pathologies that lead to cognitive and/or behavioral decline (Alzheimer's disease, dementia with Lewy bodies and the frontotemporal dementia spectrum). 

## DEFINING BIOMARKERS AND THE ROLE OF MOLECULAR NEUROIMAGING IN THE DIAGNOSIS OF NEURODEGENERATIVE DEMENTIAS

Biomarkers can be defined as measures or indicators of physiological or pathological processes or responses to a therapeutic intervention^10^. An ideal biomarker should^11^: 1) reflect a fundamental aspect of pathophysiology; 2) indicate the real presence of the pathology and not merely an increased risk; 3) exhibit high sensitivity and specificity (80% or more); 4) be effective in predicting early or pre-clinical stages; 5) allow monitoring of disease severity or rate of progression; 6) indicate the effectiveness of the therapeutic intervention and 7) be non-invasive, economically feasible and available. Although this consensus was originally proposed in the context of AD, it can also be applied to biomarkers in other neurodegenerative diseases.

The biomarkers used in neurodegenerative diseases can be schematized according to the detection methods, including cerebrospinal fluid (CSF), serum and structural and molecular neuroimaging biomarkers^12^. Molecular neuroimaging methods have gained prominence in the study of biomarkers in neurodegenerative diseases for several reasons^13^. First, the molecular neuroimaging studies allow detection of pathological changes prior to morphological changes observed in structural neuroimaging exams, such as magnetic resonance imaging (MRI). Second, new radiotracers with affinity to bind to pathological proteins, like amyloid β peptide (Aβ) plaques and tau protein, have increasingly been developed. Moreover, neuroimaging allows the study of proteinopathy, its anatomical distribution, and neuronal networks. 

Molecular neuroimaging methods are based on the use of radioisotopes (or radionuclides) for image acquisition. The basic principle is the administration of a radiotracer, a molecule with biological properties bound to the radionuclide and whose emission of the radioactive signal is detected by a scanner, forming a radiotracer^14^. There are two methods for acquiring molecular neuroimaging: positron emission tomography (PET) and single photon emission tomography (SPECT)^14^.

Currently the most used radioisotope in the SPECT neuroimaging study is technetium-99m (99mTc). Radiotracers marked with 99mTc are fat-soluble compounds that cross the blood-brain barrier and are distributed according to cerebral blood flow^14^. Therefore, most SPECT radiotracers assess brain perfusion. The advantage of SPECT is its low acquisition cost, longer half-life, greater simplicity in radiotracer synthesis and, consequently, greater availability. However, brain images acquired by SPECT have lower spatial resolution, which makes them less sensitive and specific than brain PET. Therefore, currently, the use of SPECT as a molecular neuroimaging biomarker is not recommended, except when PET is not available.

On the other hand, the most used radionuclides in PET are carbon-11 (^11^C) and fluor-18 (^18^F), which are more unstable isotopes with shorter half-lives. Therefore, PET requires radiopharmacy laboratories and production centers (cyclotrons) closer to the image acquisition center, which makes the method less available and more expensive. However, despite these disadvantages, PET images usually have a better accuracy as biomarkers of neurodegenerative diseases. Currently, semiquantitative analysis software has been incorporated into the routine analysis of PET scans, thus increasing the accuracy of the diagnostic method, and allowing better inter-examiner comparison^14^ ^,^ ^15^. Some of these software tools, such as 3D-SSP (Cortex ID Suite software, GE Healthcare or Scenium software, Siemens Healthineers), compare the examinations with a database of normal individuals, reporting the results as z-scores (or standard deviation) from the mean of normality. 

The oldest PET method is the use of ^18^F-fluorodeoxyglucose (FDG) as a radiotracer, with which it is possible to study the regional brain glucose metabolism (rBGM)^14^ ^,^ ^15^. rBGM, in turn, is an indirect measure of neuronal and glial synaptic activity^16^. Therefore, areas of neuronal and synaptic injury can be indirectly determined by regions of regional brain hypometabolism and, thus, FDG-PET plays a role as a biomarker of neurodegeneration^17^. The regional hypometabolism pattern provides neuroanatomical information on the neurodegenerative process which, in turn, has a good specificity for each type of neurodegenerative dementia (Table 1) ^18^. Not surprisingly, in several groups of neurodegenerative diseases, the pattern of hypometabolism was included in the diagnostic criteria, as will be discussed later. 

**Table 1.** Patterns of regional glucose hypometabolism on brain FDG-PET in the main groups of neurodegenerative dementias.

Neurodegenerative dementia	Variants	Typical hypometabolism pattern
Alzheimer's disease	Amnestic (or limbic)	Temporoparietal association cortex, precuneus and posterior cingulate. In more advanced cases, there is an extension to the prefrontal cortex.
	Logopenic variant of primary progressive aphasias	Temporoparietal association cortex, precuneus and posterior cingulate, asymmetrical, worse on the left
	Visual (or posterior cortical atrophy)	Temporoparietal association cortex, precuneus and posterior cingulate with occipital extension
	dysexecutive/ behavioral	Temporoparietal association cortex, precuneus and posterior cingulate with frontal extension
	Corticobasal syndrome	Temporoparietal association cortex, precuneus and asymmetric posterior cingulate, with involvement of the basal ganglia
Lewy bodies dementias	Dementia with Lewy bodies	Temporoparietal and occipital association cortex, precuneus with posterior cingulate preservation (“cingulate island sign”)
	Parkinson's disease dementia	Temporoparietal and occipital association cortex, with frontal extension, precuneus with preservation of the posterior cingulate.
Frontotemporal dementias	Behavioral variant	Dorsolateral prefrontal cortex, orbitofrontal, anterior cingulate, anterior insula, and anterior temporal regions. It may have an asymmetry, worse on the right.
	Non-fluent variant of primary progressive aphasias	Left inferior frontal region and left insula, with extension to the anterior cingulate and dorsolateral frontal region
	Semantic variant of primary progressive aphasias	Bilateral anterior temporal pole, worse on the left.
	Progressive supranuclear palsy	Dorsolateral, ventrolateral, and medial frontal cortex, including supplementary motor area and premotor cortex, caudate, thalamus, and midbrain
	4R tauopathy corticobasal syndrome (corticobasal degeneration)	Dorsolateral frontoparietal cortex, including sensorimotor cortex, thalamus, and striatum, of asymmetrical pattern

PET: Positron Emission Tomography; FDG: [^18^F] Fluorodeoxyglucose.

Despite its good accuracy, FDG-PET is still regarded as a biomarker of neurodegeneration. Therefore, the current frontiers of molecular neuroimaging techniques in cognitive decline have reached the detection of misfolded protein deposits^14^ ^,^ ^15^ ^,^ ^18^. These new methods were only possible after the development of radiotracers that bind specifically to these pathological proteins. In the following topics we will address some of these molecular neuroimaging modalities in the context of the main groups of neurodegenerative dementias. 

## MOLECULAR NEUROIMAGING IN ALZHEIMER'S DISEASE AND THE ATN CLASSIFICATION

AD is pathologically defined by the presence of senile plaques (extracellular deposit of Aβ) and neurofibrillary tangles (intraneuronal accumulation of hyperphosphorylated tau protein), in addition to signs of a neurodegenerative process (neuronal and synaptic loss, astrogliosis and microglial activation)^12^ ^,^ ^19^. The first diagnostic criteria were clinical, following the exclusion of others causes associated with cognitive decline^20^ ^,^ ^21^. However, changes in AD diagnostic criteria have been observed in recent decades as new knowledge of AD's pathophysiology and natural history have been better elucidated. Biomarkers played a vital role in these paradigm shifts. 

In 2011, a working group from the National Institute on Aging and Alzheimer’s Association (NIA-AA) developed the current diagnostic criteria for AD^6^ ^,^ ^20^. These new criteria proposed the concept of stages of disease evolution, based on the “amyloid cascade” hypothesis^9^ ^,^ ^22^. According to this model, the disease follows a continuous course that starts from a preclinical phase (defined as the absence of cognitive decline and the presence of positive biomarkers for AD pathology), followed by a stage of mild cognitive impairment (MCI) and, finally, a stage of dementia due to AD^6^ ^,^ ^20^. For the first time, the preclinical stage, and the possibility of non-amnestic AD variants (posterior cortical atrophy, logopenic variant of primary progressive aphasias and dysexecutive/behavioral variant) were admitted. 

In the 2011 criteria, the biomarkers are recommended only in selected cases: pre-senile onset dementias (< 65 years), rapidly progressive dementias, atypical dementias or for differential diagnoses with other neurodegenerative dementias. Later studies show that these 2011 clinical criteria, without the use of biomarkers, have a sensitivity of 70.9 to 87.3% and a low specificity of 44.3 to 70.8% when compared to pathological diagnosis^17^. Biomarkers have also been incorporated into the diagnosis of MCI due to AD^23^. 

More recently, in 2018, the NIA-AA proposed new criteria for the biological definition of AD based on biomarkers according to an “ATN” system, especially targeting clinical trials, in which certainty of the pathological process involved is required (Table 2) ^24^. According to the pathological process, AD biomarkers have been divided into biomarkers of amyloid pathology (A), tau pathology (T) and neurodegeneration (N), summarizing the ATN classification (Table 2) ^7^ ^,^ ^24^ ^,^ ^25^. 

**Table 2.** Alzheimer's disease biomarkers classified according to the pathological process (ATN system). Adapted from Jack et al.^28^.

Pathology	Biomarker
Amyloid Pathology (A)	Decreased Aβ42 in CSF Positive amyloid PET
Tau pathology (T)	Increased hyperphosphorylated protein in CSF Positive tau PET
Neurodegeneration (N)	Increase total Tau protein in CSF Cortical atrophy on magnetic resonance imaging Regional glucose hypometabolism on FDG-PET

CSF: cerebrospinal fluid; Aβ: beta-amyloid peptide; PET: Positron Emission Tomography; FDG: [^18^F] Fluorodeoxyglucose.

The purpose of these 2018 criteria is to apply them in clinical research, while the 2011 criteria would remain valid for use in clinical care(Table 3) ^24^ ^,^ ^26^. According to these 2018 criteria, AD is defined by the positivity of biomarkers for amyloid (A+) and tau (T+), regardless of the presence or absence of neurodegeneration (N- or +). When only Aβ (A+) is present, but without evidence of tau (T-) pathology, the denomination should be “Alzheimer's pathological changes”. It therefore represents the initial stage of AD (amyloidosis without tauopathy) according to the “amyloid cascade” hypothesis. When there is positivity for tau protein (T+) and/or for neurodegeneration (N+), but negativity for amyloid pathology (A-), the diagnosis of Alzheimer`s pathology must be excluded, and it should be called SNAP (suspected non-Alzheimer’s pathophysiology). The term "clinical AD syndrome" was reserved for the situation in which the patient meets the clinical criteria for AD (amnestic or non-amnestic variants), but there is no information on amyloid or tau biomarkers. 

**Table 3.** Diagnostic categories according to the ATN system (Amyloid-Tau-Neurodegeneration) within the continuum of the biological definition of Alzheimer's disease. Adapted from Jack et al.^28^.

ATN profile	Category according to biomarker profile
A- T- N-	Normal Alzheimer disease D biomarkers
A+ T- N-	Alzheimer's continuum
A+ T+ N-	Alzheimer's disease (without neurodegeneration)
A+ T+ N+	Alzheimer's disease (with neurodegeneration)
A+ T - N+	Alzheimer's continuum + Non-Alzheimer Pathology
A- T+ N-	Suspected non-Alzheimer’s pathophysiology
A- T- N+	Suspected non-Alzheimer’s pathophysiology
A- T+ N+	Suspected non-Alzheimer’s pathophysiology

In 2021, the International Working Group (IWG), in opposition to the 2018 NIA-AA criteria, criticized the excessive importance of the use of biomarkers for the diagnosis of AD^27^. They claim that several studies show an increased frequency of amyloidosis and neurodegeneration associated with aging even in cognitively normal individuals^8^ ^,^ ^28^ ^,^ ^29^, although the presence of amyloidosis in normal elderly is associated with higher rates of progression to cognitive decline^8^ ^,^ ^28^ ^,^ ^30^ ^-^ ^34^. Nevertheless, the IWG recommendation is that the diagnosis of AD should be restricted to people with A+T+ biomarkers and who have a cognitive syndrome compatible with AD^27^. This IWG definition, therefore, would fulfill the diagnosis of AD only in the MCI and dementia states with evidence of A+T+ biomarkers, which, on the one hand, increases specificity. On the other hand, the disease usually begins many years earlier and studies are continuously focusing on pre-clinical diagnosis and treatment, which are not possible when using the IWG recommendation. 

### Amyloid PET

Since the early 2000s, radiotracers for the detection of insoluble Aβ peptide deposited in neuritic plaques (amyloid PET) have been widely used in AD studies^15^ ^,^ ^18^. The first radiotracer developed was the Pittsburgh Compound-B labeled with carbon-11 (PIB), widely used in clinical research^13^ ^,^ ^18^. Sequentially several other Aβ peptide ligands were developed, all labeled with fluorine-18. These radiotracers (florbetapir, florbetaben and flutemetamol) are already cleared by the US regulatory agency for commercial clinical use^18^. All these radiotracers are clinically equivalent, but those linked to fluorine-18 have the advantage of a longer half-life of 110 minutes compared to ^11^C, whose half-life is only 20 minutes^14^ ^,^ ^15^ ^,^ ^35^. In practice, due to carbon-11’s low half-life, ^11^C-PIB needs to be synthesized in a cyclotron located in the same building of the imaging site^14^ ^,^ ^15^ ^,^ ^35^. But despite this disadvantage, studies with PIB have shown this to be a radioligand with a high accuracy in the detection and anatomical location of neuritic plaques. 

Studies comparing amyloid PET with post-mortem pathological diagnosis show a sensitivity of 96% and a specificity of 100% in cases of dementia due to AD^18^. Sensitivity is not 100% because all radiopharmaceuticals have high affinity for insoluble fibrillar amyloid in neuritic plaques, with good anatomopathological correlation, but they are insensitive to soluble Aβ oligomers^18^ ^,^ ^36^ ^-^ ^38^. Also, amyloid PET may be less sensitive in earlier stages of the disease^36^, mostly because of the dichotomized nature of its interpretation: scans are usually classified as positive or negative, which most commonly include individuals with mild deposition as negative. Therefore, changes in CSF Aβ may precede changes in amyloid PET. Amyloid PET is usually classified as "positive" if there is a loss of gray and white matter differentiation in at least two of the following six areas: frontal, temporal, lateral parietal, precuneus, anterior cingulate, and posterior cingulate cortex(Figure 1) ^39^ ^,^ ^40^. On the other hand, the amyloid PET image is classified as "negative" when there is a clear contrast between gray and white and no significant uptake in the cortex. In the quantitative analysis of amyloid PET, it was agreed to determine the standard uptake values ​​ratio (SUVr) of cortical areas normalized for gray matter of the cerebellum, as this is a region that does not present significant Aβ deposits in AD^39^ ^,^ ^40^. Interestingly, amyloid radiotracers have high affinity for the myelin sheath in the absence of Aβ deposits, leading to their use in the investigation of demyelinating diseases such as multiple sclerosis. 

The pattern of radiotracer uptake for amyloid in the cortex follows the pattern commonly seen in post-mortem studies, with involvement of the frontal regions, followed by the precuneus and posterior cingulate and finally areas of temporoparietal association, medial temporal region, primary cortical areas. and striatum^36^. Furthermore, as was already known, the distribution of amyloid pathology is not distinguished between AD variants, unlike tau pathology, whose neuroanatomical distribution is reflected in the clinical phenotype^41^. 

It is important to note that a positive amyloid PET alone does not fulfill the criteria for the diagnosis of AD in any of the existent criteria^6^ ^,^ ^20^ ^,^ ^24^ ^,^ ^26^, as cognitively normal individuals, with MCI, or even with other forms of dementia (particularly dementia with Lewy bodies) may have positive tests for the presence of amyloid^6^. While between 70-90% of patients clinically diagnosed with AD test positive for amyloid, about 30-40% of cognitively normal older adults over 80 years also test positive for amyloid^6^. Rates of positive amyloid PET in cognitively normal older adults vary among studies, some reporting high rates of 20% at around 65 years and 60% at 85 years^14^. A PET-based study of our group found 18% of amyloid positivity in controls (71.19 ± 6.1 years old), and 76% in clinically-defined AD, and as low as 37% of positivity in amnestic MCI (mean ages of 73.7 ± 7.3 and 73.0 ± 5.8, respectively)^42^. 

These data indicate that the process of extracellular Aβ peptide deposition is a common process in brain aging. Therefore, diagnostic criteria based on biomarkers indicate that the diagnosis of AD should show positivity for both amyloid and tau biomarkers (A+T+). On the other hand, studies show that cognitively normal older adults with positive amyloid PET have a higher chance of evolving to AD in the next 10 to 20 years, indicating that part of these subjects may be in a prodromal or preclinical stage of AD^43^ ^,^ ^44^. Indeed, in older adults below 80 years of age who have cognitive decline, a negative amyloid PET excludes the diagnosis of AD. Table 4 summarizes the advantages and dis-advantages of amyloid PET. 

**Table 4.** Advantages and disadvantages of amyloid PET.

Advantage	Disadvantages
Negative amyloid PET excludes AD; Very useful for differentiating pre-senile onset dementias; Allows you to differentiate from primary tauopathies and TDP-43pathies; Positive amyloid PET has high predictive power for conversion to AD.	Above 80 years of age, 30 to 40% of normal older adults have a positive amyloid PET; Not very useful in differentiating from alpha-synucleinopathies; High cost and little availability; Not yet incorporated into AD clinical criteria.

PET: Positron Emission Tomography; AD: Alzheimer disease; TDP-43: transactive response DNA binding protein of 43 kDa.

### Tau PET

Tau protein plays a role in the proteins that give support and stability to the microtubules that are found in axons. Hyperphosphorylation of the tau protein leads to formation of insoluble filaments, which are deposited as intracellular inclusions, ultimately leading to cell death^12^. Tau exists in six isoforms distributed in two groups in equal proportions: three isoforms have three repeats (tau 3R), and the other three have four repeats (tau 4R) of the sequence of amino acids that bind to microtubules^15^ ^,^ ^45^. AD is characterized by a tauopathy with the presence of two subtypes of tau (3R and 4R). However, according to the amyloid cascade hypothesis, AD is considered a tauopathy secondary to amyloid pathology^22^. Other 3R and 4R tauopathies are chronic traumatic encephalopathy, primary age-related tauopathy (PART) and some cases of FTD by a mutation in the MAPT gene^45^ ^,^ ^46^. 

On the other hand, primary tauopathies are divided between the 3R and 4R. 3R-tauopathies are found in some cases of FTD and can aggregate in characteristic intracytoplasmic inclusions called Pick bodies^45^ ^,^ ^46^. The group of 4R-tauopathies includes corticobasal degeneration (CBD), progressive supranuclear palsy (PSP), FTD with parkinsonism linked to chromosome 17 and most cases of the nonfluent/agrammatic variant of primary progressive aphasia (nfvPPA)^45^ ^,^ ^46^. 

Studies of tau biomarkers have shown a recent increase in interest for a few reasons: failure of clinical trials of anti-amyloid therapies, a more significant correlation between tauopathy and AD progression (unlike amyloid pathology), recent studies suggesting pathways of tauopathy progression independent of amyloid pathology and the diagnosis of primary tauopathies^46^ ^-^ ^49^. 

Since the 2010s, several ligands for tau protein detection (tau PET) have been developed and validated from post-mortem comparative studies^46^ ^,^ ^49^ ^-^ ^51^. ^18^F-FDDNP was the first radiotracer developed, however, it had low specificity, binding to neurofibrillary tangles and amyloid aggregates, which caused it to be discontinued^45^. Other first-generation radiopharmaceuticals have been developed: ^11^C-PBB3, ^18^F-flortaucipir (previously ^18^F-AV1451 or ^18^F-T807), ^18^F-THK5317, and ^18^F-THK5351 (45,46). In 2020, ^18^F-flortaucipir was the first FDA-approved for clinical and commercial use^52^. These radiotracers bind to regions typically affected regions in AD patients, such as the lateral temporal, lateral and medial parietal (precuneus) and posterior cingulate^45^ ^,^ ^46^ ^,^ ^49^ ^,^ ^51^. Studies with flortaucipir in different AD phenotypes show that the distribution of the tau protein follows the anatomical distribution associated with each cognitive manifestation of the variants^45^ ^,^ ^46^ ^,^ ^49^ ^,^ ^51^. 

Although these radiotracers have a higher affinity for tau protein compared to ^18^F-FDDNP, they still have some affinity for other protein aggregates, such as TDP-43, Aβ, and alpha-synuclein. For example, studies show that PET with ^18^F-flortaucipir (^18^F-AV1451) has regional uptake in the semantic variant of primary progressive aphasias, whose pathology is most commonly TDP-43^45^ ^,^ ^46^. Furthermore, these radiotracers have low specificity in distinguishing the type of tauopathy (3R ​​vs. 4R) and the level of maturity of the tau deposit (pre-tangle, mature neurofibrillary tangle, and phantom tangle), as well as the type of cell affected (neurons versus glia). It is known, for example, that in primary tauopathies, especially in PSP and CBD, glial cells also show deposits of hyperphosphorylated tau^45^ ^,^ ^46^. The low affinity for other non-3R/4R tauopathies limited the use of first-generation tracers in the clinic. 

The second generation of radiotracers was more recently developed. Some examples are: ^18^F-MK-6240, ^18^F-RO-948, ^18^F-PI-2620, ^18^F-GTP1, ^18^F-PM-PBB3, ^18^F-JNJ311 and ^18^F-JNJ-067^45^ ^,^ ^46^. These second-generation radiotracers have the advantage of having a higher affinity for hyperphosphorylated tau protein and less binding to non-tau targets compared to first-generation radiotracers^53^ ^-^ ^55^. Second-generation radiotracers have shown that tau PET has a good accuracy in predicting the trajectory of cognitive decline in cognitively normal subjects or those with MCI, superior to amyloid PET^50^ ^,^ ^56^. 

Two of these tracers (^18^F-MK-6240 and ^18^F-PI-2620) are in an advanced stage of testing in clinical settings and showed promising results in 4R tauopathies, such as CBD and PSP. The main advantage of this generation of tau PET is its use in primary tauopathies. PSP has the advantage of being exclusively a tauopathy (unlike CBD or even FTD which can be phenotypes of different proteinopathies). Some first-generation radiotracers show good affinities for tau deposits in other non-AD tauopathies, and the uptake pattern may indicate whether it is a primary or secondary AD tauopathy^45^ ^,^ ^49^. These radiopharmaceuticals also have an affinity for monoamine oxidase B (MAO-B), which is abundantly present in base nuclei and may lead to false positives^45^ ^,^ ^49^. ^18^F-flortaucipir, the most used first-generation tracer, cannot reliably differentiate the types of non-AD tauopathies (PSP, CBD or FTD) and may not detect the early stages of Braak (I to III)^57^ ^,^ ^58^. ^18^F-RO-948 was also shown to have good accuracy in differentiating AD from non-AD tauopathies in a comparative post-mortem study^55^. 

If second-generation tau PET tracers continuously prove their value, they have the potential to replace amyloid and FDG-PET in some scenarios in clinical practice (e.g., in the different types of tauopathies), providing “one-stop-shop” studies of pathology and disease staging at the same time.

### FDG-PET in Alzheimer's disease

Despite advances in PET ligands specific for pathological protein deposits, FDG-PET remains the main molecular neuroimaging method available in clinical practice. Studies show that a pattern of regional hypometabolism in areas of association temporoparietal, medial temporal, precuneus and posterior cingulate have a high specificity for AD ranging from 86 to 98%, compared to pathological diagnosis (Figure 1) ^17^ ^,^ ^35^. Its accuracy in the evaluation of AD and FTD variants is comparable to amyloid PET, with the advantage of providing insights on disease stage^17^. Furthermore, FDG PET findings precede the structural changes seen on MRI, another biomarker of neurodegeneration. Patients with MCI who present this “AD pattern” have a 75 to 100% accuracy in predicting conversion to dementia^35^ Interestingly, the posterior cingulate has been shown to be the region whose involvement best predicts MCI conversion to dementia due to AD^35^. 

FDG-PET is also very useful in the differential diagnosis between AD and other neurodegenerative dementia, by showing specific regional patterns of hypometabolism in each condition (Table 1). This good specificity even helps in the differential diagnosis between AD variants and other groups of neurodegenerative dementias. For example, anterior cingulate hypometabolism makes it possible to accurately differentiate a bvFTD from a dysexecutive/behavioral variant of AD^41^ ^,^ ^59^. In CBS, temporoparietal and posterior cingulate hypometabolism suggest an AD pathology and predicts a positive amyloid PET with 88.5% of accuracy and 100% of positive predictive value, whereas asymmetric hypometabolism of the dorsolateral frontoparietal cortex, including sensorimotor cortex, thalamus, and striatum, suggests non-AD CBS (generally, 4R tauopathy)^60^. 

Comparative studies of tau PET and FDG-PET imaging indicate an overlap between areas of tau protein accumulation and hypometabolism. This overlap, on the other hand, does not occur between amyloid PET and FDG PET. This has two consequences: first, that tauopathy correlates more directly with neuronal injury and synaptic dysfunction than does amyloid pathology, and second, that FDG PET may be a better substitute for tau PET than amyloid PET in places where this test is unavailable^42^ by showing simultaneously specific patterns of neurodegeneration and helping to stage the disease. This use of FDG-PET as a proxy for tau pathology was previously foreseen^26^. 

Additionally, the combination of amyloid and FDG-PET provided incredibly high sensitivity and specificity of 97% and 98%, if both are congruent, in a post-mortem study^17^. This was replicated in a PET study using the rationale of the “ATN” staging, where an FDG-PET with an AD-pattern predicted amyloid positivity in 93% of cases (27 of 29 FDG-positive individuals), and provided alternative diagnostic hypotheses (e.g. frontal hypometabolism suggestive of FTD) in amyloid-negative individuals^42^. However, it is essential to emphasize that according to the amyloid cascade hypothesis, the changes in tau PET would precede those observed in FDG-PET, a less specific biomarker of neurodegeneration.

## MOLECULAR NEUROIMAGING IN DEMENTIA WITH LEWY BODIES

Dementia with Lewy bodies (DLB), like Parkinson's disease (PD) and multiple system atrophy (MSA), is part of a group of neurodegenerative diseases characterized by the presence of alpha-synuclein neuronal inclusions^61^. DLB is described as the second cause of neurodegenerative dementia after the age of 65. The current diagnostic criteria for DLB were defined by a consensus in 2017 that included, among other points, biomarkers^62^. These biomarkers were classified as indicative, whose specificity is high enough to define the probability of DLB, and supportive biomarkers, with lower sensitivity, but with good accuracy to allow clinical suspicion. The indicative biomarkers are polysomnography, myocardial sympathetic innervation scintigraphy, and PET/SPECT with radiotracer for dopamine transporter (DAT). Supportive biomarkers included FDG-PET, MRI and electroencephalogram. None of these biomarkers define the presence of alpha-synuclein in vivo, but somehow reflect the repercussions of the pathophysiological process. 

PET or SPECT using dopamine tracers aim to assess the integrity of the nigrostriatal dopaminergic pathways through the study of dopamine transporter, a presynaptic protein with a role in dopamine uptake^63^. The most used radiotracer is 123-I-ioflupane (DATSCAN), but some countries use other tracers like TRODAT-1 (a tropane derivative labeled with ^99m^Tc), whose images are acquired with SPECT, a more spread technology than PET. In DLB and PD, there is a low uptake in basal ganglia, demonstrating the loss of dopaminergic neurons from the nigrostriatal pathways(Figure 1). The specificity and sensitivity to distinguish from AD is 90% and 78%, respectively, which would justify it as an indicative biomarker^62^. It is also very useful for differentiating neurodegenerative parkinsonism from secondary ones (such as medication), and from essential tremors. However, it would not have good specificity to distinguish from other dementia with parkinsonism (eg, PSP and CBD), which makes it questionable as an indicative biomarker in a patient exclusively with parkinsonism and dementia, without other main symptoms^64^.

Myocardial scintigraphy with iodine-123-labeled meta-iodo-benzyl-guanidine (123I-mIBG) is another biomarker with good sensitivity (69%) and high specificity (87%, reaching 94% in mild cases) to differentiate DLB from AD^62^. mIBG is a molecule captured by pre-synaptic noradrenergic neurons and is therefore a biomarker of postganglionic sympathetic innervation. In patients with DLB, there is a denervation of sympathetic fibers in the myocardium and, consequently, there is a low cardiac uptake of ^123^I-mIBG on scintigraphy. Care should be taken in diabetic patients, autonomic neuropathies, heart disease and users of tricyclics antidepressants, as there may be false-positive low uptake in these individuals.

FDG-PET is considered a supportive biomarker of DLB. As in AD, the pattern is usually of temporoparietal hypometabolism. However, unlike in AD, there may be also a characteristic occipital hypometabolism in DLB, with a sensitivity of 70% and specificity of 74% (Figure 1) when present^62^. In addition, there is a relative preservation of the posterior cingulate (which is classically affected in AD), called the “posterior cingulate island sign”^65^. Amyloid PET, in turn, is of little use in distinguishing between AD and DLB, as there is a variable frequency of deposition of amyloid pathology in patients with DLB.

## MOLECULAR NEUROIMAGING IN FRONTOTEMPORAL DEMENTIAS

Frontotemporal dementias (or frontotemporal lobar degeneration) correspond to a group of neurodegenerative diseases where there is a selective involvement of the frontal and temporal lobes^66^. From a clinical phenotype point of view, they comprise two large groups: the behavioral variant (vbFTD) and primary progressive aphasias (PPA). Among the PPAs, in turn, there are three variants: the agrammatic or non-fluent (nfvPPA) and the semantic (svPPA). The logopenic variant of PPA is not part of the FTD syndrome, because of the common underlying AD pathology. More recently, some authors propose a third group of FTD: the motor variants, which would include atypical parkinsonism (PSP and CBD) or motor neuron disease (FTD with amyotrophic lateral sclerosis)^67^. bvFTD corresponds to the second cause of degenerative dementia in the pre-senile age group, after AD.

Just as there is phenotypic heterogeneity, FTD is characterized by pathological variability (Figure 1). Three proteins are associated with FTD: tau, TDP-43 (transactive response DNA binding protein of 43 kDa) and FUS (fused in sarcoma protein). To date, there is no specific radioligand for the last two proteins. In the case of tau protein, studies focus mainly on the use of tau PET in AD, but some studies have been published in samples of patients with FTD, PSP and CBD. As previously described, PSP has been studied as a model of primary tauopathy because, unlike the other phenotypes, PSP is almost exclusively caused by deposits of 4R-tau protein^68^.

Some PET studies highlight the importance of tau protein deposition in FTD. In a study with ^18^F-flortaucipir in patients with various FTD phenotypes, including genetic forms of FTD (MAPT and C9orf72 mutations), there was an increase in uptake in the left inferior frontal gyrus compared to the right in cases of nfvPPA. Half of the cases of bvFTD had increased uptake in the frontotemporal region, being more intense in MAPT mutation carriers. Furthermore, uptake of ^18^F-flortaucipir was observed in cases of svPPA and mutation of C9orf72 mutation, whose proteinopathy is usually TDP-43^69^. In another study, using the radiopharmaceutical ^18^F-MK-6240 in patients with genetic FTD, a mild uptake was noted in cases of symptomatic MAPT carriers. Only one case of a patient with a non-tau mutation (in this case, C9orf72 mutation) showed minimal tracer uptake, suggesting that ^18^F-MK-6240 may be a potential marker for primary tauopathies^70^.

However, FDG-PET is still the most-used molecular neuroimaging method in FTD. The presence of a clinical syndrome with typical pattern of hypometabolism makes the diagnosis of FTD or PPA likely, according to diagnostic criteria^71^ ^,^ ^72^. In bvFTD, the pattern of hypometabolism is the involvement of the orbitofrontal region, dorsolateral prefrontal cortex, ventromedial prefrontal cortex, anterior cingulate, temporal poles, and basal ganglia, often asymmetrically (Figure 1). In patients whose underlying pathology is a tauopathy (particularly in Pick's disease), anterior frontotemporal atrophy may be very pronounced compared to the posterior temporoparietal cortex ("knife-edge" pattern). Patients with MAPT mutations present a relatively symmetrical pattern of hypometabolism of the orbitofrontal, dorsolateral prefrontal, and especially the anterior temporal lobes. In individuals carrying the GRN mutations, hypometabolism is asymmetric, with extension to the parietal lobe^73^. In patients with FUS pathology, there is a characteristic involvement of the caudate nuclei and ventral frontal cortex^73^ ^,^ ^74^. 

In PPAs, hypometabolism reflects the pattern of pathology involvement within the language neural network. In nfvPPA, the left inferior frontal cortex is affected, commonly extending to the anterior insula. The hypometabolism of the temporal poles, more to the left, with extension to the lateral temporal cortex, is the hallmark of the svPPA pattern. lvPPA is characterized by left temporoparietal hypometabolism, a typical pattern of AD^72^.

## WHEN AND HOW SHOULD MOLECULAR NEUROIMAGING TESTS BE REQUESTED IN COGNITIVE DECLINE IN CLINICAL PRACTICE?

**Figure 2.**
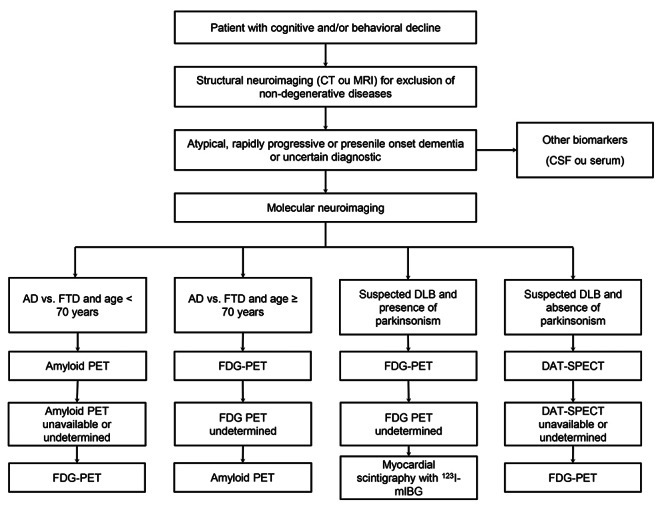
Flowchart showing a proposal in the sequence of the request of molecular neuroimaging in the diagnostic investigation of cognitive and/or behavioral decline in the clinical practice. Abbreviations: FTD: frontotemporal dementia; AD: Alzheimer's disease; DLB: dementia with Lewy bodies.; PET: Positron Emission Tomography; SPECT: single photon emission tomography; FDG: ^18^F-Fluorodeoxyglucose; DAT: dopamine transporter; ^123^I-mIBG: iodine-123-labeled meta-iodo-benzyl-guanidine; CSF: cerebrospinal fluid; MRI: magnetic resonance imaging; CT: computed tomography.

In clinical practice, molecular neuroimaging should be requested carefully, as it is a high-cost and low-availability exam. But it is very useful in situations where the diagnosis of cognitive decline is uncertain, especially when the clinical manifestations are atypical, early-onset or rapidly progressive dementias. It is important to emphasize that before requesting a molecular neuroimaging, the recommendation is always to perform a structural neuroimaging, in order to exclude structural pathologies. CSF biomarkers (in the future, plasma biomarkers) are alternatives to molecular neuroimaging. However, CSF biomarkers have also high-cost, in addition to presenting some measurement problems, especially for the beta-amyloid peptide (pre-analytical errors, lack of interlaboratory reliability) which can lead to false-results. FDG-PET is the most available molecular neuroimaging test. It is important to emphasize that FDG-PET should ideally be performed in clinics whose evaluation is not only visual, but also a semi-quantitative analysis is performed to increase the accuracy of the method. Amyloid PET is restricted to a few specialized centers and, so far, tau PET is not available in our country. Regardless of availability, FDG has a good accuracy for the differential diagnosis between neurodegenerative dementias. We propose a flowchart in the request for molecular neuroimaging in the diagnostic investigation of cognitive and/or behavioral decline (Figure 2).
